# Molecular characterisation of the emerging measles virus from Roraima state, Brazil, 2018

**DOI:** 10.1590/0074-02760180545

**Published:** 2019-03-18

**Authors:** Cátia Alexandra Ribeiro Meneses, Valdinete Alves do Nascimento, Victor Costa de Souza, Rodrigo Melo Maito, Marconi Aragão Gomes, Claudeth Rocha Santa Brígida Cunha, Ilma de Aguiar Antony, Maria Eliane Oliveira e Silva, Daniela Palha de Souza Campos, André de Lima Guerra Corado, Karina Pinheiro Pessoa, Dana Cristina da Silva Monteiro, Osnei Okumoto, Marília Coelho Cunha, Flávia Caselli Pacheco, Felipe Gomes Naveca

**Affiliations:** 1 Secretaria de Estado da Saúde de Roraima, Laboratório Central de Saúde Pública de Roraima, Boa Vista, RR, Brasil; 2 Fundação Oswaldo Cruz-Fiocruz, Instituto Leônidas e Maria Deane, Manaus, AM, Brasil; 3 Secretaria de Estado da Saúde de Roraima, Coordenadoria Geral de Vigilância em Saúde, Boa Vista, RR, Brasil; 4 Ministério da Saúde, Secretaria de Vigilância em Saúde, Brasília, DF, Brasil; 5 Ministério da Saúde, Coordenação Geral de Laboratórios de Saúde Pública, Brasília, DF, Brasil

**Keywords:** measles virus, Brazil, genotype, virus genome

## Abstract

Measles is a human infectious disease of global concern that is caused by the measles virus. In this study, we report the complete genome sequencing of one measles virus isolate, genotype D8, that was obtained directly from a urine sample in Boa Vista city, the capital of Roraima state in Brazil. Phylogenetic reconstruction grouped the genome described in this study with that of samples from Australia, South Korea, and Italy. To our knowledge, this is the first complete genome sequence of a wild-type measles virus reported from Latin America. Therefore, the present data strengthen the current knowledge on the molecular epidemiology of measles worldwide.

Measles is a highly contagious airborne disease that begins as an acute febrile illness characterised by symptoms such as fever, coryza, conjunctival hyperaemia, cough, and a maculopapular skin rash. Severe complications including blindness and life-threating morbidities such as severe diarrhoea and pneumonia may develop, particularly in immune-compromised patients. Two rare but highly severe neurological diseases, measles inclusion body encephalitis (MIBE) and subacute sclerosing panencephalitis (SSPE), may also be caused by measles virus infection.^1^

The measles virus is an enveloped, single-stranded, negative-sense RNA virus with a genome of around 16 kb that belongs to the *Morbillivirus* genus in the *Paramyxoviridae* family. A total of eight proteins, six structural (nucleoprotein, phosphoprotein, matrix protein, fusion protein, haemagglutinin, and the large polymerase) and two non-structural (V and C proteins) are encoded by the viral genome.^1^ RNA viruses are more likely to accumulate variability in their genome sequence compared to DNA viruses due to the lack of proofreading activity of RNA polymerases and the higher viral titres during the acute phase of the illness.^2^ This diversity is reflected in 24 genotypes of the measles virus known to date (A, B1, B2, B3, C1, C2, D1, D2, D3, D4, D5, D6, D7, D8, D9, D10, D11, E, F, G1, G2, G3, H1, and H2). Nevertheless, between October 2017 and September 2018, only five of these genotypes (B3, D4, D8, D9, and H1) were reported (http://www.who-measles.org/Public/Data_Mnt/who_map.php).

Measles is a vaccine-preventable disease with a decreasing mortality observed during the last decades. Since the 1980s, the total number of deaths related to measles dropped from more than 2 million per year to approximately 100,000 per year due to the improvement of social indicators (e.g., better nutrition) and global efforts to increase vaccination coverage.^1^ Nevertheless, timely surveillance of suspected measles cases with highly sensitive and specific molecular diagnostic tools combined with genetic characterisation of the isolates is of paramount importance for the global efforts to eliminate the virus.^1^ ^,^ ^3^

On 14 February 2018, the health authorities of Roraima state notified the Brazilian Ministry of Health regarding a suspected measles case in the municipality of Boa Vista, the state capital. A one-year-old non-vaccinated Venezuelan child presented with fever and a rash accompanied by cough, coryza, and conjunctivitis, which was later confirmed as a measles case in the laboratory.

In August 2018, the Central Laboratory of Roraima state (LACEN-RR) started testing samples from suspected measles cases using the real-time polymerase chain reaction (PCR) protocol developed by the Centers for Disease Control and Prevention (CDC), USA. The procedures followed annex 6.2 of the Manual for the Laboratory-based Surveillance of Measles, Rubella, and Congenital Rubella Syndrome (3rd edition, June 2018, http://www.who.int/immunization/monitoring_surveillance/burden/laboratory/Annex_6.2.pdf?ua=1). Until 21 January 2019, a total of 579 suspected measles cases were reported in Roraima state, 332 in the capital Boa Vista, and 226 in other 14 municipalities across the state. Among the reported cases, 355 were confirmed as measles, leading to an incidence rate of 80/100,000 inhabitants. Eleven Brazilian states have confirmed measles cases, totalling 10,302 cases countrywide.^4^

As requested by of both Roraima state and the Brazilian health surveillance authorities, we selected one positive sample with a high viral load (Ct 22.0) to submit to a protocol for amplification of the entire genome and nucleotide sequencing. Initially, we aligned all measles D8 genomes with MAFFT v7.388.^5^ The consensus sequence was used to design primers spanning the entire genome of measles D8 with the aid of Primal software^6^ covering spans of around 400 bp. Two other primers targeting the initial 28 bases of the 5’ untranslated region (UTR) and the final 25 bases of the 3’ UTR were designed with a modified version of Primer3 v2.3.7 embedded in the Geneious software v10.2.6 (Biomatters, Auckland, New Zealand).^7^ This primer design may be used for both next-generation sequencing, as previously described for the Zika virus,^6^ and capillary sequencing performed in the present study. Primer sequences and details of the reverse transcription polymerase chain reaction (RT-PCR) protocol used for entire genome amplification and nucleotide sequencing are listed in Supplementary data (Table, Fig. 1).

Briefly, four overlapping amplicons encompassing the entire measles genome were generated using the Superscript IV One-Step RT-PCR System (ThermoFisher Scientific, Waltham, MA, USA). Amplicons were precipitated with molecular biology grade polyethylene glycol (PEG) 8000 and used as a template for nucleotide sequencing with BigDye terminator v3.1 in an ABI 3130 genetic analyser (ThermoFisher Scientific).

A total of 63 trace files were trimmed for quality and used for assembly employing the Geneious’s map-to-reference tool and the National Center for Biotechnology Information (NCBI) measles reference sequence (GenBank accession number NC_001498.1). The final whole genome sequence of MVs/Roraima.BRA/31.18[D8] isolate was found to contain 15,894 nucleotides with no ambiguity or unidentified (N) bases, a Q40 score of 99.8%, and a GC content of 47.4%.

Initially, the sequence reported here was genotyped using both the nucleoprotein (N) gene and the hemagglutinin (H) gene with the measles genotyping tool available at http://www.who-measles.org/Public/Tool/genotype_tool.php to confirm the D8 genotype. We subsequently conducted a maximum likelihood phylogenetic analysis with PhyML software^8^ using the N gene region of the Roraima sample, the genotypes reference sequences available in the Measles Nucleotide Surveillance (MeaNS) database (http://www.who-measles.org/), and four other sequences representing the genotype D8 lineages (MVi/Villupuram.Ind/03.07, GenBank accession number: FJ765078.1; MVi/Hulu-Langat.MYS/26.11, JX486001.1; MVs/Frankfurt_Main.DEU/17.11, KF683445.1; MVs/Republic_of_Komi.RUS/35.13, KT588030.1). This approach confirmed that the Roraima sample belongs to the lineage MVi/Hulu-Langat.MYS/26.11 [Supplementary data (Fig. 2)].

We next used the Basic Local Alignment Search Tool (BLAST) to conduct a database search employing the MegaBLAST algorithm against the entire non-redundant nucleotide collection (nr/nt). The first 5 closest matches returned in the search were samples from Geoje, South Korea (MF496201, score: 29146 bits, 15857/15894 identical sites), Brisbane, Australia (MH638233, score: 29137 bits, 15834/15862 identical sites), Seoul, South Korea (MF496200, score: 29130 bits, 15854/15894 identical sites), Changwon, South Korea (MF496202, score: 29035 bits, 15837/15894 identical sites), and Ancona, Italy (MH173047, score: 29000 bits, 15816/15872 identical sites).

Finally, we analysed the sequence obtained in this study in combination with a dataset containing all 60 complete measles genome sequences belonging to the genotype D8 that were available in GenBank on 25 January 2019 with the jModelTest program (version 2.1.7 v20150220) and computed likelihood scores for 5 nucleotide substitution schemes (40 models). The GTR+I model was selected based on the Akaike information criterion (AIC) of the jModelTest program and subjected to Bayesian phylogenetic reconstruction with 2 runs and 30 million Markov chain Monte Carlo (MCMC) generations using the MrBayes software (version 3.2.6).^9^ All MrBayes analyses were performed on the Cyberinfrastructure for Phylogenetic Research (CIPRES) Science Gateway (version 3.3, https://www.phylo.org). This analysis returned two main clades. One clade [main clade I, highlighted in blue in Figure and Supplementary data (Fig. 3)] contained 31 sequences from the United Kingdom (2012-2013), one from the Netherlands (2013), one from Canada (2010), one from Germany (2013), five from Vietnam (2014), three from India (2010, 2014, and 2016), and a sequence from Texas, USA (2007) as the outgroup. The second clade [main clade II, highlighted in red in Figure and Supplementary data (Fig. 3)] contained the same five sequences returned in the BLAST analysis with the highest scores obtained for those from South Korea (2016), Australia (2015), and Italy (2017), as well as other sequences from India (2016-2017), United Kingdom (2012), California, USA (2010), and Virginia, USA (2009), with this last one representing the outgroup of this clade. The Bayesian tree grouped the isolate MVs/Roraima.BRA/31.18[D8] with a high posterior probability (1.0) in this second clade, in close proximity to the samples from Australia, South Korea, and Italy returned in the BLAST search (Figure).

**Figure:**
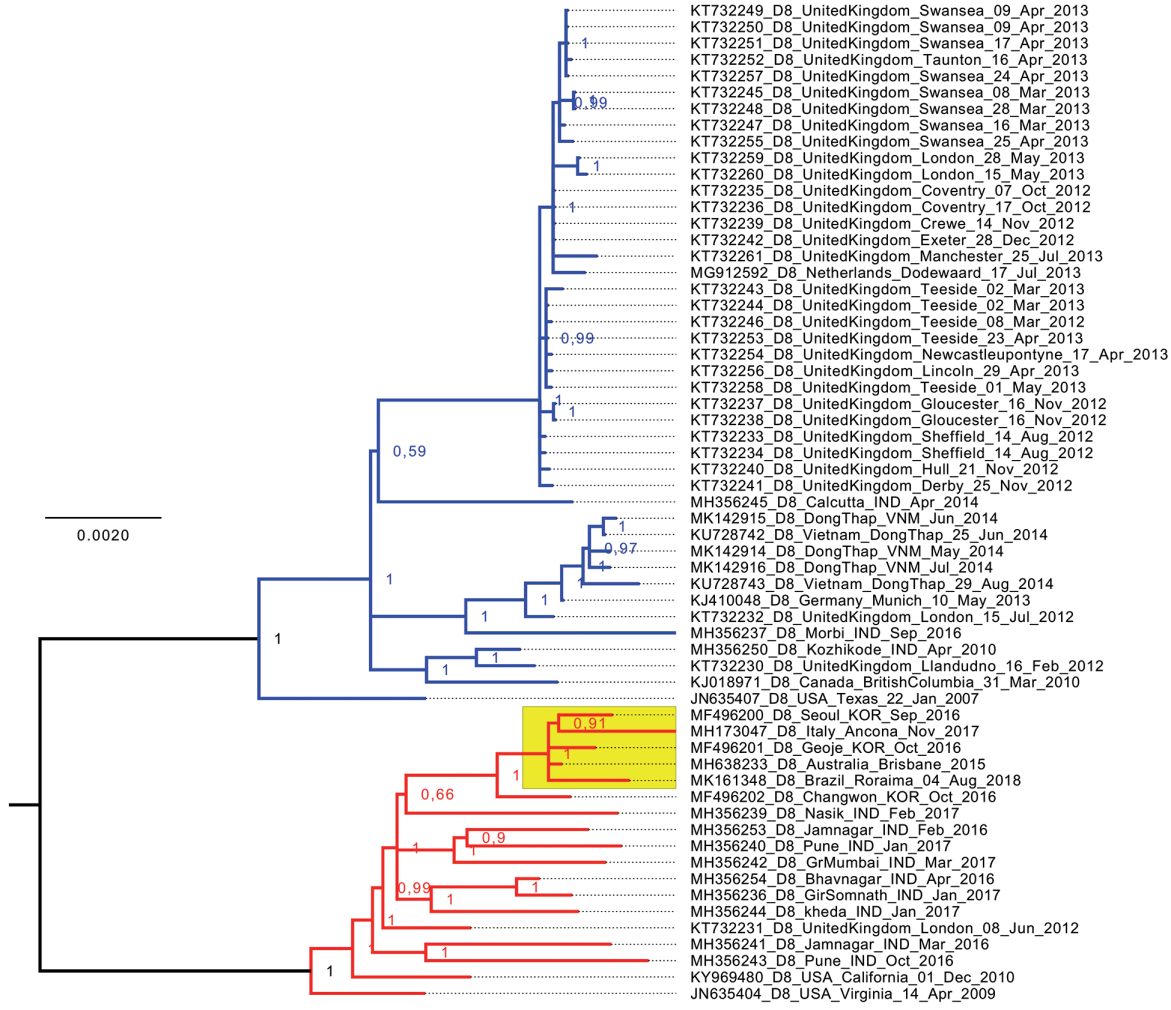
Phylogenetic tree of complete measles virus genomes. A mid-rooted Bayesian tree with increasing node order was constructed with the MrBayes software (version 3.2.6) and 61 taxa (complete coding sequence and intergenic regions ranging from position 108 to 15,785 for reference sequence NC_001498.1) representing all complete measles genotype D8 genomes available in GenBank on 25 January 2019 and the sequence reported in this study. Posterior probability values of each node are shown. The clade containing the sequence described in this study is highlighted in yellow. The scale bar represents nucleotide substitutions per site.

Surprisingly, we did not find any record of a complete genome sequence of the measles virus from Latin America in the public databases including GenBank, Virus Pathogen Resource (ViPR, http://www.viprbrc.org), and MeaNS. Therefore, we compared the phylogenetic signal of the 450 bp fragment of the N gene with complete genome sequences using the same dataset [Supplementary data (Fig. 3)]. Overall, the main clades I (blue) and II (red) were maintained, albeit with a lower resolution within each clade that prevented a more in-depth analysis of the genetic relationships. For example, one strongly supported clade (posterior probability = 1.0) encompassing sequences KJ018971 (GenBank accession number, Canada), KT732230 (United Kingdom), and MH356250 (India) within the main clade I of the phylogenetic tree of complete genomes is missing in the phylogenetic tree generated with the N gene 450 bp fragment. Moreover, the sequence from Virginia, USA (JN635404, 2009) may be considered an outgroup of the entire clade II in the phylogenetic tree of complete genomes (posterior probability = 1.0). Nevertheless, when the N gene 450 bp dataset was used for phylogenetic reconstruction, this sequence was directly grouped with a sequence from India (MH356239, 2017).

Furthermore, a BLAST analysis conducted on 24 January 2019 with the N gene 450 bp fragment commonly used for genotyping returned 181 sequences with 100% identity to the sequence described in this study. These sequences were obtained from samples collected in 15 different countries worldwide: Australia (2013, 2014, 2015, 2016, 2017), Belgium (2018), China (2016), Colombia (2018), Germany (2017), Italy (2014), Japan (2017), Malaysia (2016), Netherlands (2016), New Zealand (2017), Oman (2017), Sweden (2017), United Kingdom (2016), USA (2017, 2018), and Venezuela (2017). Therefore, analysis of this fragment alone may not suffice for robust phylogeographic analysis.

Although the resolution of the phylogenetic tree built with the N gene 450 bp dataset decreased, we conducted a second phylogenetic analyses using this genomic region and all measles D8 genotype sequences available in GenBank on 25 January 2019. This search returned 3,280 nucleotide sequences and 3,107 sequences containing the N gene 450 bp fragment were selected for further investigation. Sequence redundancy was reduced by using the Cluster Database at High Identity with Tolerance (CD-HIT) program (http://weizhong-lab.ucsd.edu/cdhit-web-server/cgi-bin/index.cgi). Thus, a final dataset containing 567 sequences was analysed with jModelTest as described above. A Bayesian phylogenetic tree was built using the MrBayes software with the GTR+I+G model for 50 million (MCMC) generations, or until the convergence hit an average standard deviation of split frequencies below 0.009.

The main conclusion of the phylogeny inference analysis using the N gene 450 bp dataset was that the sequence described in this study does not cluster with the sequences obtained during the previous measles outbreak in Pernambuco, Brazil in 2013 [GenBank accession number: KF738753; clade highlighted in green in the Supplementary data (Fig. 4)]. This result suggests that the measles viruses causing the outbreaks in Brazil in 2013 and 2018 did not have the same origin. The lack of available nucleotide sequences of other D8 measles samples (e.g., Ceará, Brazil, 2015) unfortunately hinders a more detailed analysis of the local transmission of this measles genotype in Brazil and Latin America.

Previous studies in Brazil have primarily used a small fragment of 450 bp of the measles N gene for genotyping.^10^ ^,^ ^11^ ^,^ ^12^ Although these studies have undoubtedly contributed to the characterisation of the molecular epidemiology of measles, sequencing of larger genome fragments or ideally complete viral genomes may further our understanding of the epidemic dynamics of any emerging or remerging viral disease.^13^ ^,^ ^14^

*Nucleotide sequence accession number* - The complete genome sequence of the MVs/Roraima.BRA/31.18[D8] isolate is available in GenBank since 20 November 2018 under the accession number MK161348.
