# Supplementary material for: Molecular characterisation of the emerging measles virus from Roraima state, Brazil, 2018
Source: Mem Inst Oswaldo Cruz. 2019 Mar 18;114:e180545. doi: 10.1590/0074-02760180545 (PMC6423661; doi:10.1590/0074-02760180545)
Supplement: Supplementary file 1 [file 1678-8060-mioc-114-e180545-s.pdf]

TABLE

Primers designed to amplify and sequence the entire genome of measles genotype D8

Primer name	Sequence	Pool	Length	Tm	GC%	Start	End
FN_MVD8_1_LEFT	TGGCCACACTTTTAAGGAGCTT	1	22	60.42	45.45	1	23
FN_MVD8_1_RIGHT	AATCCCTCAGGGTCTTGCACTT	1	22	61.64	50.00	488	466
FN_MVD8_2_LEFT	TCAAGAGGTACCAACATGGAGGA	2	23	60.76	47.83	351	374
FN_MVD8_2_RIGHT	TTCGGCAATCCTAGGTTTGTTCC	2	23	61.12	47.83	756	733
FN_MVD8_3_LEFT	GGAGAGAAAATGGTTGGATGTGGT	1	24	61.05	45.83	632	656
FN_MVD8_3_RIGHT	ATCGACCAAAGTTCAAACCCCC	1	22	61.08	50.00	1063	1041
FN_MVD8_4_LEFT	TGCACCCTATATGGTAATCCTGGAG	2	25	61.52	48.00	926	951
FN_MVD8_4_RIGHT	ACTTAATCTGCTGGACCCGGTT	2	22	61.69	50.00	1380	1358
FN_MVD8_5_LEFT	TTCTACACGGTGATCAAAGTGAGAA	1	25	60.22	40.00	1255	1280
FN_MVD8_5_RIGHT	GCTGTGTGGACCTGGTTCCTAA	1	22	62.20	54.55	1660	1638
FN_MVD8_6_LEFT	AAAGTTCAGACACGGACACCCC	2	22	62.70	54.55	1522	1544
FN_MVD8_6_RIGHT	TCGTCATCGCTCTCTCCAGATC	2	22	61.38	54.55	1965	1943
FN_MVD8_7_LEFT	ATCAGACAACCCAGGACAGGAC	1	22	61.68	54.55	1815	1837
FN_MVD8_7_RIGHT	CAGAAGCCCTGAACCCCATAGA	1	22	61.49	54.55	2240	2218
FN_MVD8_8_LEFT	TCAGGCCTTGATGGTGATAGCA	2	22	61.49	50.00	2095	2117
FN_MVD8_8_RIGHT	TACCAGATTCGGGTGTCCACTC	2	22	61.47	54.55	2570	2548
FN_MVD8_9_LEFT	TGCAACCCAATGTGCTCGAAAG	1	22	62.22	50.00	2448	2470
FN_MVD8_9_RIGHT	CGGGATTGAGTTCGACATCTGC	1	22	61.62	54.55	2876	2854
FN_MVD8_10_LEFT	GCAGATCAACAAGCAAAATATCAGCA	2	26	61.12	38.46	2751	2777
FN_MVD8_10_RIGHT	GGCGAGATCGTTAGCTCCTTTG	2	22	61.37	54.55	3184	3162
FN_MVD8_11_LEFT	AAAAAGATGAGCTCAGCCGTCG	1	22	61.50	50.00	3028	3050
FN_MVD8_11_RIGHT	AACAACCCCCAGCAGAAACATG	1	22	61.61	50.00	3507	3485
FN_MVD8_12_LEFT	CAAAGGGTCGATCGCTCCGATA	2	22	62.21	54.55	3371	3393
FN_MVD8_12_RIGHT	TAAACAACACGGAACCTCTGCG	2	22	61.37	50.00	3806	3784
FN_MVD8_13_LEFT	ACCCCACTAACTCTCCTCACAC	1	22	61.09	54.55	3678	3700
FN_MVD8_13_RIGHT	AATGTGAAGACTGGTGCCTCCT	1	22	61.63	50.00	4110	4088
FN_MVD8_14_LEFT	ATGGTCCACATCGGGAACTTCA	2	22	61.69	50.00	3981	4003
FN_MVD8_14_RIGHT	TCTGTGGAGTCTTTTGGAGGGG	2	22	61.62	54.55	4431	4409
FN_MVD8_15_LEFT	AATGATGACCAAGGACTATTCAAAGTTC	1	28	60.12	35.71	4305	4333
FN_MVD8_15_RIGHT	AAGTTTTGTTTAGTTTGCCGGGG	1	23	60.43	43.48	4767	4744
FN_MVD8_16_LEFT	GGAAAGAAACCCCCAGCAACTG	2	22	61.92	54.55	4615	4637
FN_MVD8_16_RIGHT	TGGTCTGGATTCTGGTTCGGTT	2	22	61.62	50.00	5049	5027
FN_MVD8_17_LEFT	ATACACACCCGACAGAACCCAG	1	22	61.99	54.55	4780	4802
FN_MVD8_17_RIGHT	TGTGGGATGCTGATGTCGTTTG	1	22	61.45	50.00	5213	5191
FN_MVD8_18_LEFT	CGCAGAAAGGAAAGGCCACAAT	2	22	61.98	50.00	5085	5107
FN_MVD8_18_RIGHT	CTACCCTCGTGCAGTTGTTGAG	2	22	61.18	54.55	5564	5542
FN_MVD8_19_LEFT	GGGGCAATCTCTCTAAGATAGGGG	1	24	61.61	54.17	5429	5453
FN_MVD8_19_RIGHT	TGGACACCCTGAACAGCCAATA	1	22	61.63	50.00	5886	5864
FN_MVD8_20_LEFT	CGGTATTGCACTTCACCAGTCC	2	22	61.51	54.55	5748	5770
FN_MVD8_20_RIGHT	ACGTGAGTTATCCGGGCCTTTA	2	22	61.48	50.00	6165	6143
FN_MVD8_21_LEFT	TATCCAGGCTTTGAGCTATGCG	1	22	60.55	50.00	6039	6061
FN_MVD8_21_RIGHT	ACGAGTGTACGAGCACAAGACT	1	22	61.64	50.00	6462	6440
FN_MVD8_22_LEFT	AGTCATCGTGCACTTTCATGCC	2	22	61.76	50.00	6341	6363
FN_MVD8_22_RIGHT	TCTGGTCCGATGACTCCAACAA	2	22	61.35	50.00	6797	6775
FN_MVD8_23_LEFT	ATCCAGACGCTGTGTACTTGCA	1	22	62.24	50.00	6659	6681
FN_MVD8_23_RIGHT	GGGAAGCCGGAGACAATTTCAG	1	22	61.52	54.55	7108	7086
FN_MVD8_24_LEFT	TCCTATGTAAGGTCACTCTGATCCT	2	25	60.10	44.00	6982	7007
FN_MVD8_24_RIGHT	ACGTCCTTAACCTGATGCTCGA	2	22	61.46	50.00	7435	7413
FN_MVD8_25_LEFT	TCTGAGCTTGATCGGGTTGCTA	1	22	61.47	50.00	7300	7322
FN_MVD8_25_RIGHT	TCTGATTGTAGTGGGCCCTGAG	1	22	61.48	54.55	7748	7726
FN_MVD8_26_LEFT	CAGATGTGGCTGCTGAAGAACT	2	22	60.87	50.00	7626	7648

Primer name	Sequence	Pool	Length	Tm	GC%	Start	End
FN_MVD8_26_RIGHT	CCACCATGCAGTTGCTGAAGTC	2	22	62.28	54.55	8031	8009
FN_MVD8_27_LEFT	TTGTCACAACTGAGCATGCACC	1	22	61.90	50.00	7898	7920
FN_MVD8_27_RIGHT	GGAAGCATGTCTCCATTCGCAA	1	22	61.52	50.00	8310	8288
FN_MVD8_28_LEFT	CCCCCTATCAACGGATGATCCA	2	22	61.36	54.55	8173	8195
FN_MVD8_28_RIGHT	AGTCCTCGCCTGCTTCCTTAAT	2	22	61.48	50.00	8643	8621
FN_MVD8_29_LEFT	GCTGACTATCCCGCCAATGAAG	1	22	61.38	54.55	8521	8543
FN_MVD8_29_RIGHT	CCACCATCCCAGAGTGAGTGAT	1	22	61.49	54.55	8964	8942
FN_MVD8_30_LEFT	GTTGCCTATAAAAGGGGTCCCC	2	22	60.69	54.55	8830	8852
FN_MVD8_30_RIGHT	CGGTGCTTGATGTTCTGACACA	2	22	61.38	50.00	9281	9259
FN_MVD8_31_LEFT	ACCCTGAAGTTCACCTAGATAGCC	1	24	61.42	50.00	9157	9181
FN_MVD8_31_RIGHT	GAAGGGCTCAAACCATTGGGAG	1	22	61.47	54.55	9621	9599
FN_MVD8_32_LEFT	CAATGCCTGAGGGACACTAACT	2	22	60.03	50.00	9501	9523
FN_MVD8_32_RIGHT	CGAGTGCAGGGAAGAAACCATC	2	22	61.51	54.55	9943	9921
FN_MVD8_33_LEFT	TGATGTATTGTGATGTCATAGAGGGGA	1	27	61.35	40.74	9811	9838
FN_MVD8_33_RIGHT	ACATTTTCAGCAGCCGTTACCG	1	22	62.01	50.00	10235	10213
FN_MVD8_34_LEFT	AGGTACTTATCATGATTTAATTGAAGCCCT	2	30	61.04	33.33	10097	10127
FN_MVD8_34_RIGHT	ATCCCATTCCCTTTGGAGAGCA	2	22	61.44	50.00	10557	10535
FN_MVD8_35_LEFT	TGAGCAGTGTGTTGACAACTGG	1	22	61.26	50.00	10424	10446
FN_MVD8_35_RIGHT	AGACAGCTAGAGTGTGGAGTGC	1	22	61.72	54.55	10906	10884
FN_MVD8_36_LEFT	ATGAGGGCATGCCAAGTGATTG	2	22	61.54	50.00	10782	10804
FN_MVD8_36_RIGHT	TTCAAGCCTCTTATGCAGCCAC	2	22	61.20	50.00	11229	11207
FN_MVD8_37_LEFT	CACAACTGATCTCAAGAAGTACTGCC	1	26	61.77	46.15	11105	11131
FN_MVD8_37_RIGHT	ACTCTAGCAGCTTCCCGTTTCT	1	22	61.41	50.00	11522	11500
FN_MVD8_38_LEFT	GGACCATCAGCACCATTCCCTA	2	22	61.82	54.55	11365	11387
FN_MVD8_38_RIGHT	CGGTCATAACCTCTCTCGATGC	2	22	60.61	54.55	11780	11758
FN_MVD8_39_LEFT	TGGGCTACTTGTGTCCCAATCA	1	22	61.63	50.00	11642	11664
FN_MVD8_39_RIGHT	CCCCCGGTTGTTGTGTCATTAC	1	22	61.71	54.55	12088	12066
FN_MVD8_40_LEFT	AGCAGGCTGTTTGTCAGAAACA	2	22	60.95	45.45	11961	11983
FN_MVD8_40_RIGHT	CCGAATCAGGCCTTTTGTGGTA	2	22	60.88	50.00	12387	12365
FN_MVD8_41_LEFT	GCATTCCTCATGGACAGGCATA	1	22	60.49	50.00	12264	12286
FN_MVD8_41_RIGHT	GTTGACTGATCCACACTCGCAG	1	22	61.49	54.55	12684	12662
FN_MVD8_42_LEFT	CATATGTGGGCAAGGCTAGCTC	2	22	61.13	54.55	12552	12574
FN_MVD8_42_RIGHT	GAGTGCTACGATCCCTCAACCT	2	22	61.53	54.55	13012	12990
FN_MVD8_43_LEFT	GATAGCTCTTGGAACGAGGCCT	1	22	61.86	54.55	12882	12904
FN_MVD8_43_RIGHT	TACATAGCTCTGCCCTCAGCTC	1	22	61.34	54.55	13306	13284
FN_MVD8_44_LEFT	TCGACTCGAGAAAGATACCGGA	2	22	60.42	50.00	13169	13191
FN_MVD8_44_RIGHT	GCTGCACACTGGCCTAAGTAGA	2	22	62.31	54.55	13601	13579
FN_MVD8_45_LEFT	GGTAACAAAATTTGAGAAGGACCATATGA	1	29	60.34	34.48	13466	13495
FN_MVD8_45_RIGHT	CGGTACTACATCCTCGTCGCTT	1	22	61.88	54.55	13929	13907
FN_MVD8_46_LEFT	TGCTCAAAATTTGCACACAACTGT	2	24	60.86	37.50	13796	13820
FN_MVD8_46_RIGHT	AGCGAGGGCGTCAAAAATGAAT	2	22	61.46	45.45	14214	14192
FN_MVD8_47_LEFT	CGTCAGGGTCTTCGTGGAACAT	1	22	62.55	54.55	14080	14102
FN_MVD8_47_RIGHT	CACCCAAGAACAAGCCGTCTTC	1	22	61.95	54.55	14491	14469
FN_MVD8_48_LEFT	CGCTAATTATGAAATCCATGCTTTCCG	2	27	61.51	40.74	14363	14390
FN_MVD8_48_RIGHT	GATGGCTGCCAATTCCTCTAGC	2	22	61.39	54.55	14826	14804
FN_MVD8_49_LEFT	CGTGGGTAGGCAGTATAGATTGC	1	23	60.86	52.17	14689	14712
FN_MVD8_49_RIGHT	GCGTCTCCCACAATTGCTTGTA	1	22	61.45	50.00	15149	15127
FN_MVD8_50_LEFT	AGCTAACCGGCTAATGAACCCT	2	22	61.49	50.00	15014	15036
FN_MVD8_50_RIGHT	CCCAAAATTTGCGGGTGATCCT	2	22	61.73	50.00	15439	15417
FN_MVD8_51_LEFT	TTAACGGACCCAAACTGTGCAAA	1	23	61.43	43.48	15229	15252
FN_MVD8_51_RIGHT	AATCAGGGCACTGTATCCGACT	1	22	61.22	50.00	15669	15647
FN_MVD8_5UTR	ACCAAACAAAGTTGGGTAAGGATAGATC	-	28	61.4	39.3	1	28
FN_MVD8_3UTR	ACCAGACAAAGCTGGGAATAGAAAC	-	25	61.3	44.0	15894	15870

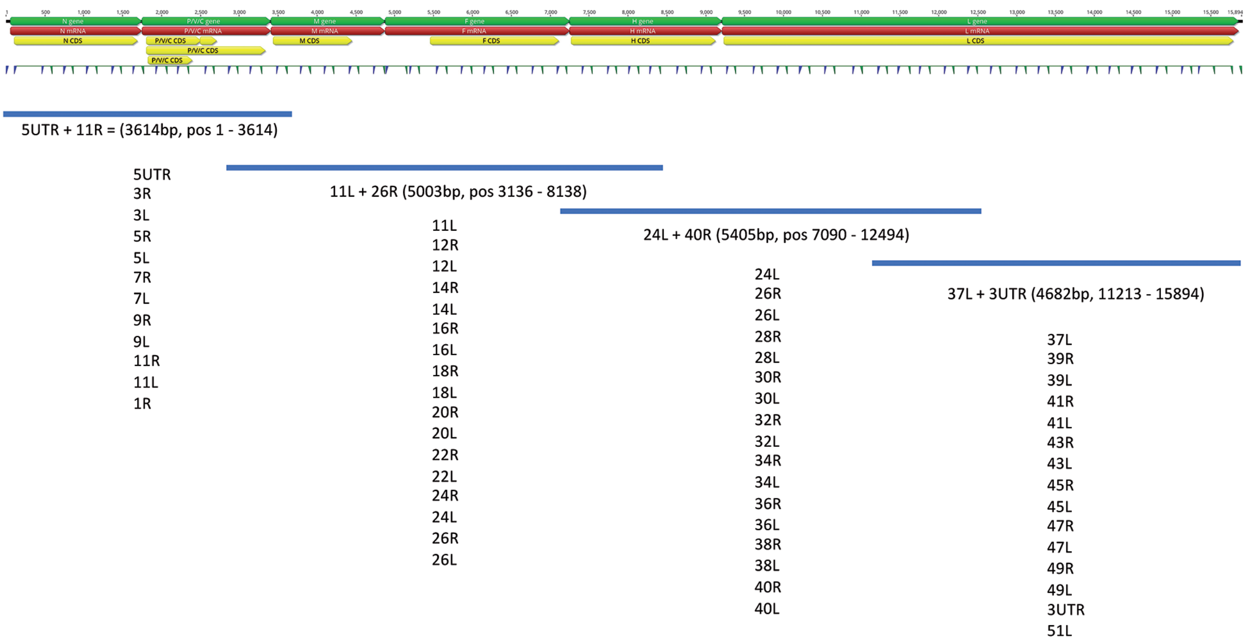

Fig. 1: primers sets used to amplify and sequence the entire measles genome. Four amplicons were generated: amplicon 1 (primers 5'UTR + 11R, 3,614 bp), amplicon 2 (11L + 26R, 5,003 bp), amplicon 3 (24L + 40R, 5,405 bp), and amplicon 4 (37L + 3UTR, 4,682 bp). Each amplicon was sequenced with the primers listed below each scheme.

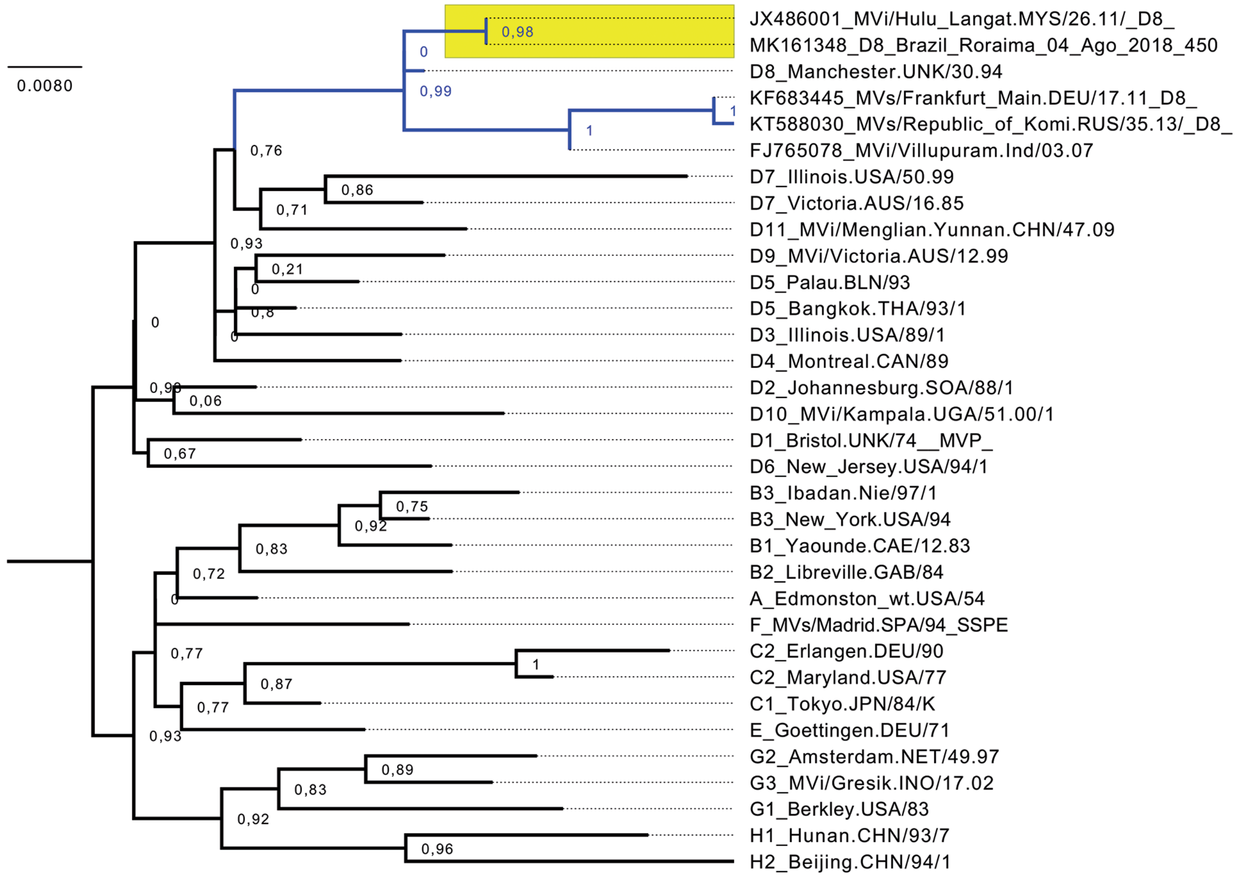

Fig. 2: phylogenetic tree of measles genotypes based on the N gene. A mid-rooted maximum likelihood tree with increasing node order was constructed with the PhyML online server (http://www.atgc-montpellier.fr/phyml/). All 24 genotypes and the 4 lineages of genotype D8 are represented. The D8 sequences are grouped in the blue clade, whereas the clade containing the Roraima sample is highlighted in yellow. The scale bar represents nucleotide substitutions per site.

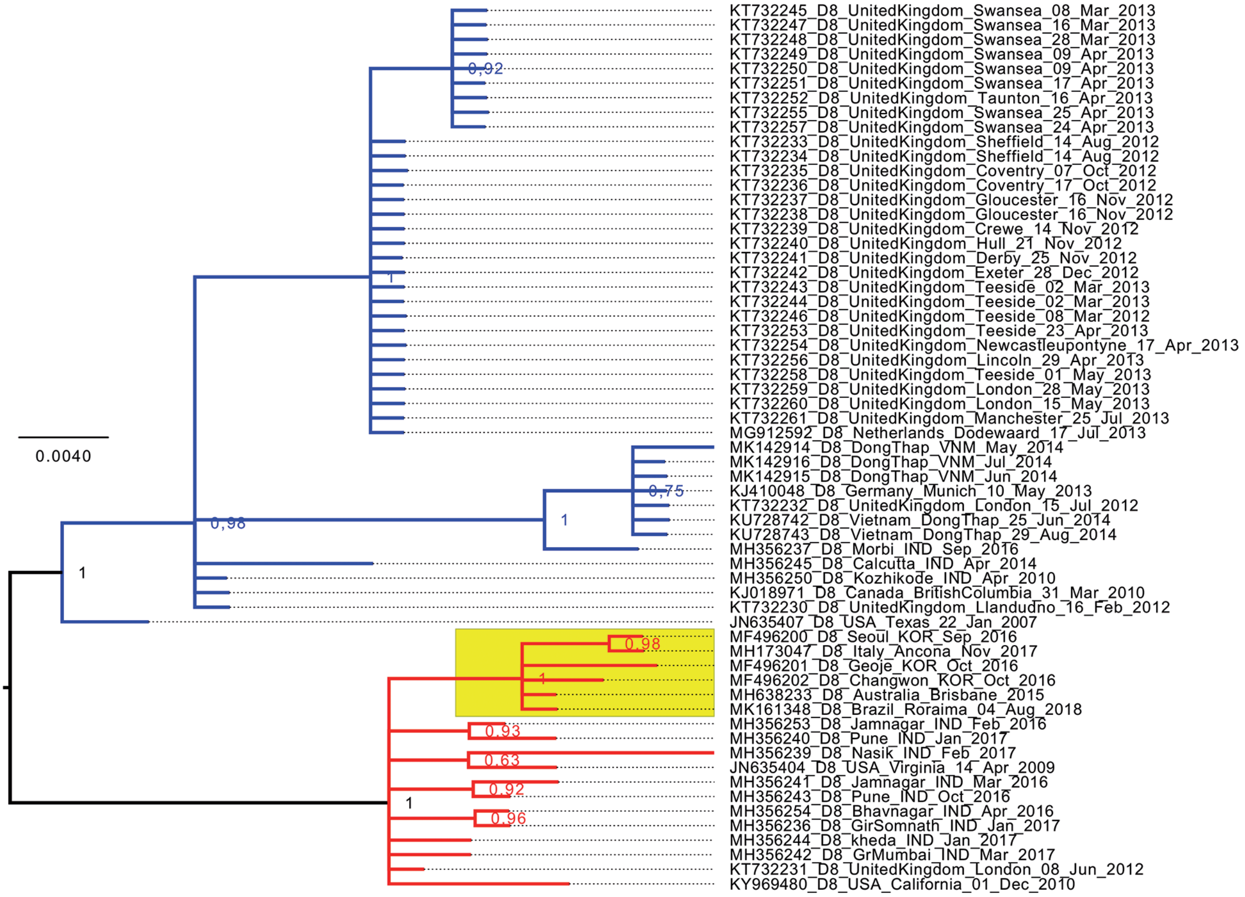

Fig. 3: phylogenetic tree of the measles virus based on the N gene. A mid-rooted tree with increasing node order was constructed using the MrBayes program (version 3.2.6). The same dataset used to analyse complete genomes (Figure) was employed for comparison. The scale bar represents nucleotide substitutions per site.

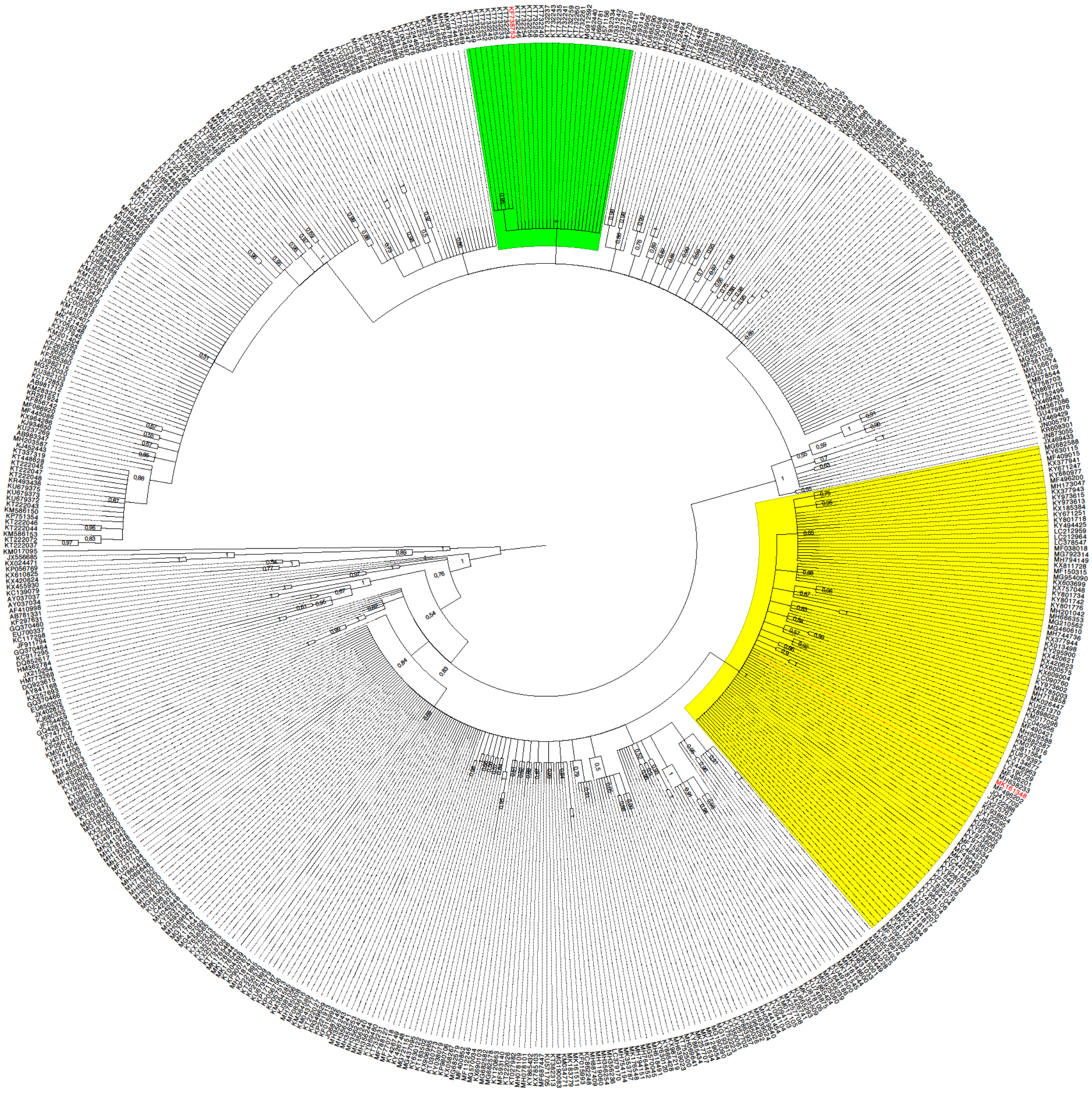

Fig. 4: phylogenetic tree of all measles genotype D8 sequences. A total of 567 non-redundant sequences, containing the 450 bp fragment of the N gene commonly used for genotyping, were used for phylogenetic reconstruction with the MrBayes program (version 3.2.6). A mid-rooted tree with increasing node order was edited with FigTree (version 1.4.3). Two clades containing sequences from Brazil are highlighted in green (GenBank accession number: KF738753, Pernambuco, 2013) and yellow (MK161348, Roraima, 2018), supporting at least two recent and different events of measles D8 introduction in Brazil since 2013.
